# DMN-seq enriches DNA hypomethylated regions for biomarker discovery using 5-methylcytosine glycosylase

**DOI:** 10.1186/s13059-026-03991-6

**Published:** 2026-02-11

**Authors:** Yiding Wang, Yang Li, Chang Ye, Iryna Irkliyenko, Lu Gao, Marc Bissonnette, Qing Dai, Weixin Tang, Chuan He

**Affiliations:** 1 Committee on Genetics, Genomics & System Biology, The University of Chicago, Chicago, IL USA; 2 Howard Hughes Medical Institute, The University of Chicago, Chicago, IL USA; 3 Pritzker School of Molecular Engineering, The University of Chicago, Chicago, IL USA; 4 Department of Chemistry, The University of Chicago, Chicago, IL USA; 5 University of California, Berkeley–University of California, San Francisco, USA; 6 Department of Medicine, The University of Chicago, Chicago, IL USA; 7 Department of Biochemistry and Molecular Biology, The University of Chicago, Chicago, IL USA; 8 Institute for Biophysical Dynamics, The University of Chicago, Chicago, IL USA

**Keywords:** DNA 5-methylcystosine sequencing, DNA hypomethylation sequencing, Cancer detection, Biomarker discovery, Low-input DNA, Cell-free DNA

## Abstract

Various methods have been developed for 5-methylcytosine (5mC) sequencing; however, effective ways to enrich hypomethylated DNA regions have been limited. Here, we describe the DEMETER-assisted 5-Methylcytosine Nicking sequencing (DMN-seq) utilizing 5mC-specific glycosylase DEMETER to nick DNA at 5mC sites, enabling 5mC detection at the single-base resolution. Leveraging this nicking activity to deplete hypermethylated sites, we adapt DMN-seq to preferentially enrich and investigate hypomethylated regions in colorectal cancer samples. When applied to cell-free DNA as low as 0.1 ng, DMN-seq significantly expands the scope of cancer biomarkers by capturing hypomethylated regions, with high sensitivity and reproducibility even in low-input clinical samples.

**Supplementary Information:**

The online version contains supplementary material available at 10.1186/s13059-026-03991-6.

## Background

DNA 5-methylcytosine (5mC), methylation of the fifth position in cytosine, one of the most important epigenetic modifications conserved in various organisms [1], plays crucial roles in transcriptional regulation, chromatin organization, and genomic stability [2]. An extensive array of proteins, including DNA methyltransferases for installation and maintenance of 5mC marks, and ten-eleven translocation (TET) proteins for stepwise oxidation of 5mC to 5-hydroxymethylcytosine (5hmC), 5-formylcytosine (5fC), and 5-carboxycytosine (5caC), facilitating dynamic turnover of 5mC. Coupled with thymine-DNA glycosylase (TDG) and enzymes in base excision repair (BER) pathway, 5mC can be converted back to cytosine (C) via the active demethylation pathway [3].

Various technologies developed to profile and detect 5mC [4] can be classified as mutation- or enrichment-based approaches (Additional file 1: Fig. S1a). As the gold standard for 5mC sequencing, bisulfite sequencing (BS-seq) distinguishes 5mC by chemically deaminating unmethylated cytosines [5]. However, harsh bisulfite conditions cause severe DNA damage. In addition, bisulfite-free enzymatic deamination methods, including Enzymatic Methyl-seq (EM-seq) [6, 7] and TET-assisted pyridine borane sequencing (TAPS) [8], utilize TET enzymes to induce base conversion and detect 5mC at the single-base level. Despite these innovations and reported successes in 5mC detection, there are limitations such as: (i) base conversion reduces read complexity and complicates mapping; (ii) incomplete conversion may generate false positives or DNA damage, limiting their applications to low input samples; and (iii) a lack of 5mC enrichment demands high sequencing depth and cost. On the other hand, enrichment-based methods, such as Methylated DNA Immunoprecipitation Sequencing (MeDIP-seq) [9, 10], selectively capture methylated DNA to reduce sequencing costs. However, they exhibit bias toward hypermethylated regions and lack single-base resolution, along with the issue of potential batch variations of 5mC antibodies. Moreover, the enrichment-based methods typically require a large amount of input materials and lack quantification accuracy.

To overcome these limitations of the current 5mC sequencing methods, we report here DEMETER-assisted 5-Methylcytosine Nicking sequencing (DMN-seq), an innovative approach that utilizes DNA glycosylase DEMETER (DME) to specifically nick DNA at 5mC sites. Originally found in plants, DME is a bifunctional DNA glycosylase that facilitates active 5mC removal [11, 12]. DME specifically excises 5mC via β- and δ-elimination in all sequence contexts to create an apurinic/apyrimidinic (AP) site through its glycosylase activity and nicks DNA with AP lyase activity [13]. Moreover, by cleaving only one strand of the target double-stranded DNA harboring symmetrically methylated sites [14], DME generates single-strand breaks (SSBs) specific to 5mC locations and proportional to 5mC density within a methylated region. Relying on these unique properties of DME, we devised DMN +, a unique experimental design for DMN-seq denoting capture of positive methylation signals. We show that DMN + enables 5mC detection at single-base resolution through employing designed adaptors that ligate only 5mC-containing fragments generated from the DME-mediated excision for subsequent sequencing. Based on the adaptor ligation position, the exact 5mC sites can be accurately mapped.

More interestingly, DMN-seq can enrich hypomethylated DNA regions through depletion of hypermethylated regions; we termed this modified experimental design DMN–, reflecting its focus on unmethylated or negative signals. Numerous 5mC biomarkers have been identified for diagnosis, prognosis, and treatment response in many cancer types, such as lung [15], breast [16], prostate [17], and colon [18, 19]. Most of these cancer-specific biomarkers tend to be promoter hypermethylation in tumor suppressor genes [20, 21], leaving hypomethylated regions, particularly promoters and enhancers for oncogenes, less investigated for mechanistic elucidations as well as their potential use as biomarkers. Global DNA hypomethylation is one of the earliest molecular events observed in early tumorigenesis [22, 23]. Through the demethylation and depression of regions such as repetitive elements, retrotransposons, and gene deserts, which comprise half of the human genome, DNA hypomethylation significantly drives the development of genome instability, a fundamental hallmark of carcinogenesis [24, 25]. Furthermore, DNA hypomethylation closely correlates with activation of cancer invasion and metastasis genes as well as drug resistance genes, contributing to tumor progression and treatment resistance [26, 27].

The enrichment of DNA hypomethylated regions using DMN–, thus, enables discovery of novel disease pathways as well as biomarkers in hypomethylated regions. We applied DMN– to genomic DNA (gDNA) from 6 sets of colorectal cancer (CRC) and adjacent healthy tissues and demonstrated its robustness in identifying 5mC sites at base resolution in hypomethylated DNA regions. We further applied DMN– to cell-free DNA (cfDNA) and demonstrated its high sensitivity and accuracy in detecting hypomethylated regions with inputs as low as 0.1 ng.

## Results

### DMN-seq detects DNA 5mC at single-base resolution

**Fig. 1:**
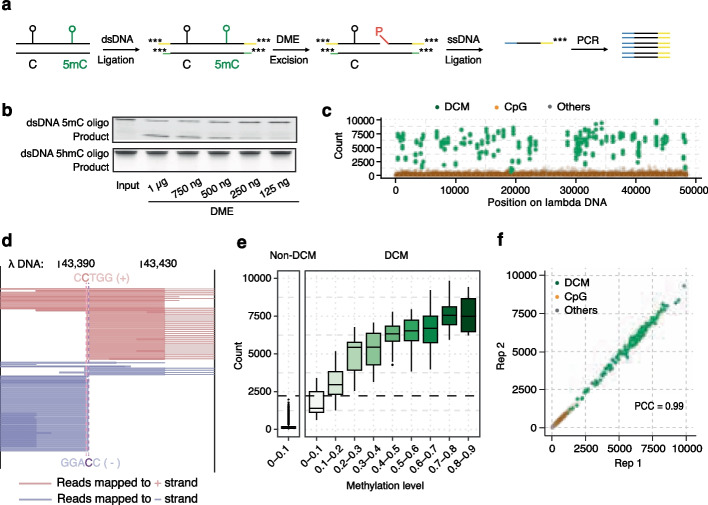
DMN-seq enables quantitative 5mC profiling at single-base resolution. **a** Schematic illustration of DMN-seq for methylated DNA enrichment. Fragmented DNAs are ligated to annealed double-stranded adaptors with 5’-phosphorothioate modifications, and subjected to sequential enzymatic treatments in order to remove all open 5’-phosphates on the remaining adapters. Subsequent treatment with DME specifically excises 5mC residues, generating new open 5’phospahtes available for ligation to customized single-stranded adaptors. Only 5mC-containing strands are amplified using primers complementary exclusively to adaptors ligated at 5mC excision sites, ensuring selective enrichment of methylation-specific fragments in the final sequencing library. **b** DNA excision efficiency of DME measured using 82 bp dsDNA oligo containing a single 5mC site and 82 bp dsDNA oligo containing a single 5hmC site. **c** Application of DMN-seq to partially methylated λ-DNA, in which all DCM motifs (CCTGG, green) are partially methylated at varying levels. The number of reads starting exactly at the 5mC sites was counted as the “enrichment intensity”. Unmethylated sites, shown for CpG (orange) and non-CpG (gray), represent the background noise level. **d** Example of reads spanning a pair of 5mC sites (positions 43,404 and 43,406) on λ-DNA. Pink and purple dashed lines highlight methylation sites on the positive and negative strands, respectively. **e** Distribution of enrichment intensity with the methylation levels at each site on DCM motif identified by UBS-seq in partially methylated λ-DNA samples. **f** Correlation of enrichment intensity between two technical replicates for each site in the partially methylated λ-DNA sample. Sites are colored by motif type. Pearson correlation coefficient (*R*) is 0.99

DME is known to nick 5mC on one of the target strands and generates a fragment containing a 5’-phosphate (5’-P). Based on this unique activity of DME, we designed adaptors and PCR primers to allow only DNA fragments with 5’-P to be ligated and amplified via PCR to construct sequencing libraries, specifically mapping the 5mC sites nicked by DME. As shown in the workflow of DMN +, the experimental design for DMN-seq to detect 5mC at single-base resolution (Fig. 1a), fragmented DNA is first ligated with customized adaptors compatible with next-generation sequencing (NGS) and attached with phosphorothioates at the end. The ligated DNA is then subjected to enzymatic digestion to remove un-ligated DNA fragments, excessive adaptors, and partially ligated DNA fragments containing 5’-P which may cause false positives. Successfully ligated DNA fragments should be protected from enzymatic digestion because of phosphorothioate modifications. After DME excision, only 5mC-containing DNA fragments should have one strand with an open 5’-P to be ligated to customized 5’-adaptors. Finally, indexing PCR is conducted using primers complementary to adaptors ligated only to 5mC-containing fragments, resulting in 5mC information being amplified and enriched in the library.

To test our experimental design, we first expressed DME and confirmed its enzymatic activity via a synthetic 82 bp dsDNA containing one 5mC site. As shown in Fig. 1b, DME excised DNA at the known 5mC site in a dose-dependent manner. Because many existing 5mC detection methods cannot distinguish between 5mC and 5hmC, leading to potential misinterpretation of data [28], we also evaluated DME's enzymatic activity on 5hmC. We used a synthetic 82 bp dsDNA oligo identical in sequence to the 82 bp 5mC dsDNA oligo but containing 5hmC in place of 5mC. DME exhibited no reactivity toward 5hmC regardless of enzyme input level, confirming its specificity for 5mC and underscoring a key advantage of DMN-seq in distinguishing 5mC from 5hmC (Fig. 1b). We then constructed sequencing libraries using partially methylated λ-DNA supplemented with two types of synthetic dsDNA oligos containing known 5mC sites as positive controls. The known 5mC sites in both synthetic spike-in oligos could be accurately detected via DMN + (Additional file 1: Fig. S2a-b). As λ-DNA is partially methylated at the DCM motif (internal C at CCAGG and CCTGG [29]), DMN + effectively distinguished all methylated DCM sites from non-DCM sites (Fig. 1c, d). We further generated a control library via Ultrafast Bisulfite Sequencing (UBS-seq) as the gold standard for the methylated sites and their methylation levels in λ-DNA. We observed a high correlation between results generated from DMN + and UBS-seq, confirming that DMN + accurately identified methylated sites across different methylation levels (Fig. 1e). Moreover, the high reproducibility between technical replicates verified the robustness of DMN + in 5mC detection (Fig. 1f). These results demonstrate the high accuracy of DMN + in quantitative 5mC profiling while minimizing false positives.

**Fig. 2:**
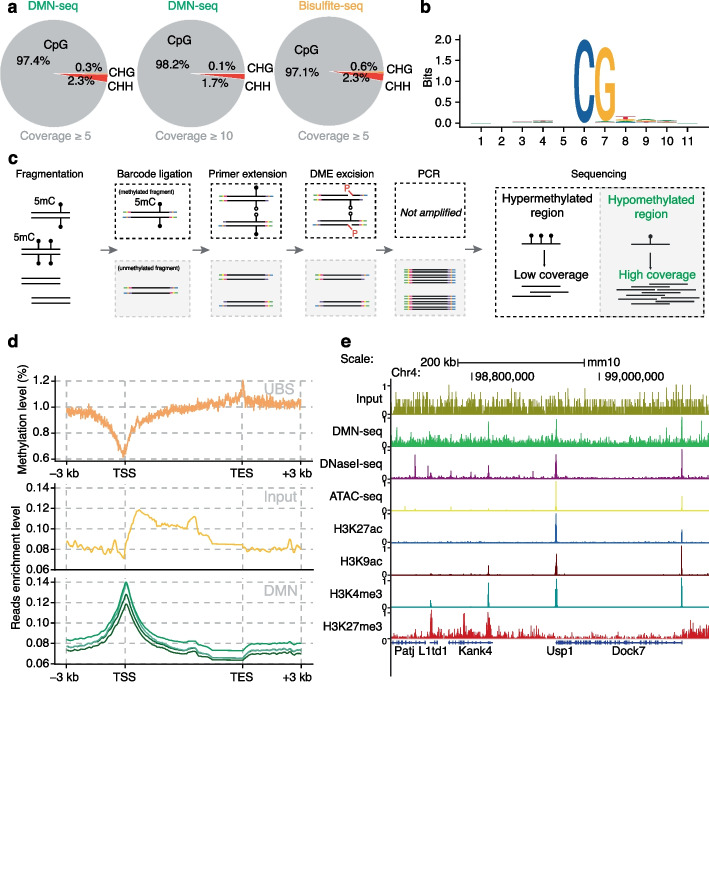
DMN-seq precisely targets methylated sites in mESCs genomic DNA and can be adapted for hypomethylation profiling. **a** CpG, CHG, and CHH sites detected by DMN-seq. At a signal coverage threshold ≥ 5, over 97% of detected 5mC sites correspond to CpG motifs, comparable to results obtained by bisulfite sequencing. **b** Consensus motif analysis for methylation sites identified by DMN-seq (*N* = 3937). **c** Schematic overview of DMN-seq for unmethylated DNA enrichment. Fragmented DNA samples ligated to individual unique barcodes allow the pooling of samples for subsequent primer extension and DME treatment. DME excision removes 5mC-containing fragments; their extended strands lacking P7 adaptors fail to amplify during the final PCR step using P5 and P7 primers, thereby enriching the sequencing library for unmethylated DNA. **d** Metagene analysis highlighting the enrichment of hypomethylated regions around transcription start sites (TSS) and transcription end sites (TES) in mESC. TSS hypomethylation is demonstrated by UBS-seq (top), whereas the input control sample does not exhibit enrichment at this region (middle). DMN-seq shows that the enrichment of methylation level at TSS ~ twofold higher than that at TES region (top), consistent with methylation level reported by UBS-seq (top). **e** Genomic snapshot illustrating DMN-seq read distribution alongside selected histone modifications, DNaseI-seq, and ATAC-seq data at a representative locus in the mESC genome. DMN-seq signals align closely with open chromatin regions and histone marks associated with enhancers and promoters,. (ENCODE data sources: DNaseI-seq ENCFF150FRY; ATAC-seq ENCFF450ZSN; H3K27ac ENCFF559BZQ; H3K9ac ENCFF165WXP; H3K4me3 ENCFF715DZB; H3K27me3 ENCFF335WEP)

To further validate the detection efficiency of DMN +, we constructed libraries using mouse embryonic stem cell (mESC) gDNA supplemented with partially methylated λ-DNA and 200-bp synthetic DNA oligos as internal controls. Shallow sequencing of approximately 10 million reads highlighted robust enrichment and accurate detection of known 5mC sites in synthetic DNA oligos with minimal background noise (Additional file 1: Fig. S3a). All 5mC sites detected in λ-DNA also presented strong DCM motifs, further confirming the accuracy of DMN + (Additional file 1: Fig. S3b). Additionally, when the 5mC sites detected in mESC gDNA were analyzed, a high correlation was observed between replicates, confirming the reproducibility of DMN + (Additional file 1: Fig. S3c). By overlapping the results with those generated using BS-seq [28], DMN + again efficiently identified methylated sites at different methylation levels. Moreover, more than 97% of the detected 5mC sites were CpG motifs, which is comparable to that from BS-seq (Fig. 2a). Motif analysis further revealed a strong CpG signal at the detected sites and motif-independent cleavage at nearby sites (Fig. 2b). These results thus demonstrate that DMN + ensures a minimal false-positive rate even with shallow sequencing depth.

### DMN-seq induces enrichment at hypomethylated regions

Given the inherent nature of DME in active DNA 5mC nicking, we reasoned that this activity could be used to detect hypomethylated DNA regions. Densely methylated regions would be subjected to multiple nicking by DME, leading to DNA fragmentation and depletion through size selection. We could in principle selectively detect 5mC sites at hypomethylated regions. With this in mind, we modified DMN-seq to deplete hypermethylated DNA regions and enrich hypomethylated DNA regions, thereby establishing the experimental design DMN–. As depicted in Fig. 2c, DNA is first ligated with unique barcodes, enabling pooling of different samples to simplify the workflow and minimize sample loss. Primer extension is then conducted on the pooled sample using only the primer complementary to the Illumina Read 2 sequence. This allows 5mC-containing strands to be excised through DME treatment, leaving their newly synthesized, complementary strands in the sample. Finally, primers complementary to P5 and P7 sequences were used for PCR. While C-containing fragments contain all the components required for exponential amplification, the complementary strands of the 5mC-containing strands lack P7 sequence. Thus, 5mC information is depleted in the final PCR, resulting in hypomethylated DNA enriched in the library.

To validate our design principle, we applied DMN– to mESC gDNA supplemented with the same internal controls. Compared with the untreated samples, all the DME-treated samples presented a minimal presence of 200 bp and a significant reduction in the number of methylated sites in λ-DNA (Additional file 1: Fig. S4a). These results suggest the high efficiency of DME treatment in depleting hypermethylated DNA while enriching hypomethylated DNA. The robustness of DMN– was further confirmed using mESC gDNA: compared with the UBS-treated sample, which served as a standard mESC methylome profile, the DME-treated samples demonstrated high enrichment of hypomethylated regions around the transcription start site (TSS) and removal of hypermethylated regions near the transcription end site (TES) (Fig. 2d). Furthermore, we compared the mESC methylation profile generated by DMN– with publicly available data from the UCSC genome browser, including ATAC-seq, DNaseI-seq, and ChIP-seq data for enhancer- and promoter-associated histone marks (H3K27ac and H3K4me3) (Fig. 2e). We observed strong overlap between regions enriched by DMN– and open chromatin regions mapped by these assays, verifying the accuracy and specificity of DMN– in enriching hypomethylated regions. We next benchmarked the performance of DMN– against Methylation-sensitive Restriction Enzyme digestion sequencing (MRE-seq) [30], another enrichment-based method for profiling unmethylated CpGs, and UBS-seq in mESC gDNA repetitive regions. DMN– exhibited higher mapping ratio in both genome-wide repetitive regions (Additional file 1: Fig. S5a) and LINE-1 elements (Additional file 1: Fig. S5b) compared to both MRE-seq and UBS-seq, with high reproducibility between duplicates. In regions flanking ± 2 kb of TSS, both MRE-seq and DMN– showed decreased enrichment with increasing 5mC levels, confirming their capacities to enrich unmethylated CpGs (Additional file 1: Fig. S5c). In particular, DMN– showed a steeper decline in enrichment as methylation increases, indicating higher specificity for truly unmethylated regions and greater efficiency in excluding partially or fully methylated DNA. These results further support the high resolution and specificity of DMN– for hypomethylation enrichment and detection.

### DMN-seq robustly maps hypomethylated regions in colorectal cancer

Colorectal cancer (CRC), the third most common malignant tumor in the world [31], has been studied extensively on diagnostic cfDNA methylation biomarkers [32, 33], including the FDA-approved *SEPT9* [18, 19] and *BMP3* [19], which are hypermethylated in the promoter regions for transcription silencing in cancerous tissues. However, global DNA hypomethylation was detected in the early stage of CRC [34], leading to demethylation of mobile genetic elements such as long interspersed nuclear element 1 (LINE-1) [35] that have been suggested for early-onset detection and progression monitoring of CRC [36–38]. Genome-wide cell-free DNA methylome profiling has verified the predictive and prognostic significance of hypomethylated LINE-1 as well as several hypomethylated genes in CRC [39–41]. Thus, exploring the clinical potential of hypomethylated regions as biomarkers for disease diagnosis, prognosis, and treatment response monitoring can be important.

**Fig. 3:**
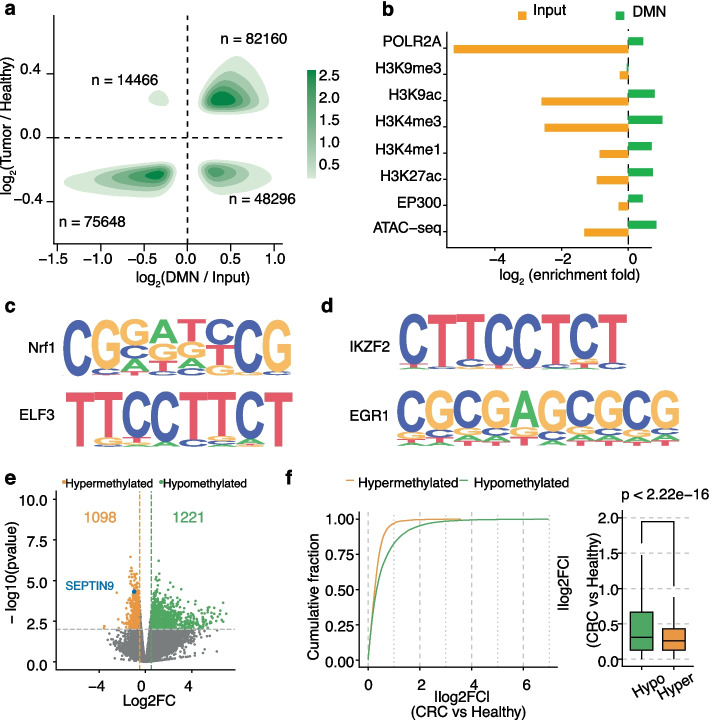
DMN-seq identifies hypomethylation pattern and tumor-specific motifs in CRC. **a** Scatter plot displaying hypomethylated regions (2000 bp segments) significantly enriched in CRC compared to healthy samples. **b** ENCODE ChIP-seq data for active histone markers (H3K4me3, H3K27ac) and transcription activators (EP300, POLR2A), along with ATAC-seq data, demonstrating enrichment of DMN-seq-detected hypomethylated regions (green) in actively transcribed and open chromatin regions, compared to input samples (yellow). DMN-seq peaks represent hypomethylated regions identified by comparing DME-treated samples to input, whereas input peaks represent hypermethylated regions identified in the inverse comparison. **c**, **d** Significant sequence motifs identified by HOMER motif searches within consensus motifs of healthy and CRC samples (**c**), and CRC-specific motifs (**d**). **e** Volcano plot illustrating differentially methylated regions within 2000 bp upstream of TSS in paired male samples. The red dot marks SEPTIN9, a known hypermethylation biomarker in CRC. **f** Distribution of the absolute log_2_ fold-change (log_2_FC) values comparing hypomethylated and hypermethylated regions in CRC samples, indicating significantly higher log_2_FC values in hypomethylated regions and reflecting abnormal upregulation of genes in CRC

We carried out a proof-of-concept study by applying DMN– to the gDNA of paired primary tumor tissues and adjacent normal-appearing colonic mucosa from 6 patients newly diagnosed with nonmetastatic CRC at the University of Chicago Medical Center (UCMC) (Additional file 1: Table S2). Using data from Reduced Representation Bisulfite Sequencing (RRBS) conducted on the same samples as standards for methylation level, we demonstrated that DMN– effectively detected differentially methylated regions (DMRs) and recapitulates expected methylation patterns, with high DMN signal corresponding to low methylation and vice versa across both CRC tumor and healthy tissues (Additional file 1: Fig. S6a, b). With an average of 55.2 million sequencing reads per sample, DMN– enabled robust detection of hypomethylated regions flanking 2 kb of each TSS, revealing a substantial enrichment of such regions in tumor compared to healthy tissues (Fig. 3a). By aligning DMN– data to ChIP-seq data for histone markers and transcriptional activators as well as ATAC-seq data, we observed high enrichment of hypomethylated regions detected in DME-treated samples in regions marked with active transcription and open chromatin (Fig. 3b). This verifies that DMN– preferentially enriches and detects hypomethylated regions. Additionally, we used the program HOMER [42] to identify sequence motifs enriched in hypomethylated regions detected in tumor and healthy tissues. For example, consistent with previous reports [43, 44], binding motifs of Nrf1, a known methylation-sensitive transcription factor (TF) that only binds to unmethylated DNA sequences [43, 45], were enriched in hypomethylated regions detected in both tissue types. Similarly, the binding motifs of ELF3, a prognostic biomarker in CRC [46], were also enriched in the consensus motifs of both groups (Fig. 3c**)**. As for TFs enriched in the detected CRC-specific motifs (Fig. 3d**)**, both EGR1 [47] and IKZF2 [48] have been found linked to disease progression with the potential of serving as CRC prognosis biomarker. These results further validate the capacity of DMN– in detecting functionally relevant hypomethylated regions in CRC, indicating its utility in uncovering novel cancer-associated biological pathways and biomarker candidates.

To assess the correlation of the detected DMRs with gene expression, we further identified 2,319 DMRs in the male group (Fig. 3e) and observed more hypomethylated regions (1,222) than hypermethylated ones (1,097) in tumors than in healthy tissues, alongside a significant reduction in average genomic methylation in CRC. These results are consistent with previous reports of CRC global hypomethylation [22, 49], a well-documented hallmark of CRC tumorigenesis due to chromosomal instability and proto-oncogene activation [24]. We next calculated the methylation percentage of the corresponding promoter regions for these DMRs (TSS + 2 kb). Tumor gDNAs tend to harbor more hypomethylated promoters than hypomethylated promoters when compared with healthy controls (Fig. 3f), as a result of global chromosomal instability during CRC development.

**Fig. 4:**
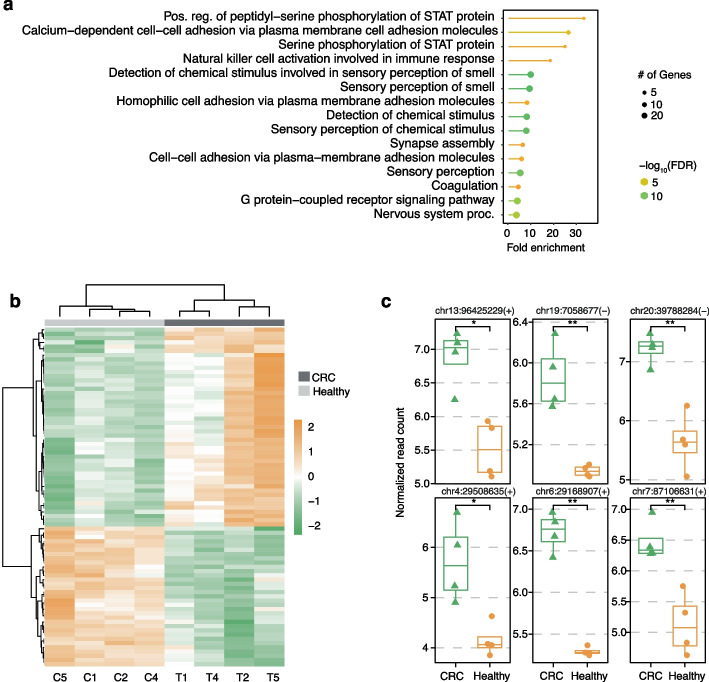
DMN-seq reveals global hypomethylation and potential hypomethylated biomarkers in CRC. **a** Gene ontology (GO) analysis illustrating biological processes enriched among genes associated with hypomethylated regions. **b** Heatmap depicting the most significantly differentially expressed regions identified in paired male samples. Regions exhibiting significant changes in normalized read counts (*p* < 0.0001) were proposed as putative CRC biomarkers. Normalized read counts (*n* = 80 regions) were scaled by z-score and clustered based on expression similarity. **c** Comparison of the top six differentially methylated regions between paired male CRC and healthy tissue samples. Genomic coordinates for each region are indicated. *P*-values (t-test) reflect the significance of methylation differences between tumor and healthy genomic DNA (gDNA)

Functional enrichment analysis of genes in hypomethylated regions in CRC revealed pathways known to be associated with CRC tumorigenesis (Fig. 4a), such as those related to transducer and activator of transcription (STAT) proteins. STAT family proteins are phosphorylated and highly expressed in CRC tissues compared with adjacent normal mucosa [50, 51] because of their roles in the Janus kinase (JAK)-STAT pathway whose dysregulation has been widely reported in CRC to contribute to cancer cell proliferation and immune evasion [52, 53]. Moreover, pathways related to sensory perception were also enriched in hypomethylated regions in CRC. Sensory perception pathways sense volatile organic compound (VOC) profile that may reflect alterations in metabolism caused by diet, gastrointestinal infection, or disease development in CRC [54, 55]. Various studies have shown significant differences in exhaled and fecal VOC profiles between CRC patients and healthy controls [56, 57]. The enrichment of sensory perception pathways revealed by DMN– further verified their upregulation in CRC. Notably, these results provide insights into hypomethylated genes, such as those associated with VOCs, as candidates as biomarkers for CRC diagnosis.

We further analyzed the hypomethylated regions from paired male CRC and healthy samples (Fig. 4b) and uncovered additional hypomethylated loci as candidates for future validation (Fig. 4c). *MBD3L3* was identified as particularly noteworthy due to its known involvement in heterochromatin formation and pathogenesis of various cancers including CRC where it has been reported as a potential prognosis biomarker [58]. Another significant gene, *OR2J2*, belongs to the olfactory receptor (OR) which has been investigated for its potential in both prognosis and alternative treatment for CRC [59]. The remaining hypomethylated loci predominantly reside within pseudogene regions, where hypomethylation may indicate genomic instability and leakage expression, contributing to CRC development and progression. Additionally, as the LINE-1 elements have been reported for its diagnostic and prognostic potential [40, 41], we further analyzed the hypomethylated regions that contain LINE-1 from all paired CRC and healthy samples (Additional file 1: Fig. S7a), and identified putative hypomethylated loci as candidates for future validation and further investigation (Additional file 1: Fig. S7b).

### DMN-seq demonstrates high detection sensitivity with input amounts as low as 0.1 ng

**Fig. 5:**
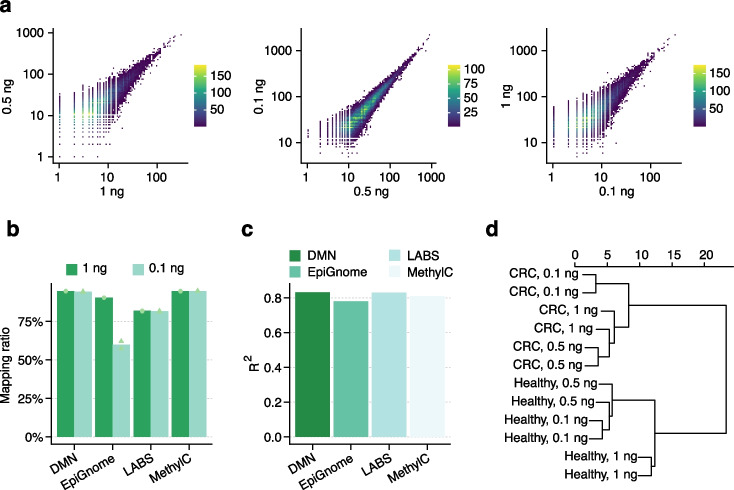
DMN-seq demonstrates high sensitivity with low-input cfDNA. **a** Scatter plot illustrating the similarity of DMN-seq signals at cfDNA input levels of 1 ng, 0.5 ng, and 0.1 ng. **b**, **c** Comparison of mapping ratios (**b**) and correlation coefficients (**c**) among DMN-seq, EpiGnome (Illumina TruSeq DNA methylation method), LABS-seq, and MethylC-seq. Correlation coefficients were calculated using the Pearson correlation based on the average methylation level in a 2000-bp sliding window across the genome, including only sites with depth ≥ 5 **d**, Cluster dendrogram indicating that clinical differences between CRC patients and healthy controls are greater than differences arising from input amounts

Methylation of cfDNA, which comprises small circulating DNA fragments of approximately 150 bp found in the bloodstream and other bodily fluids [60], has attracted extensive interest in recent years as a powerful noninvasive biomarker for cancer diagnosis, monitoring, and prognosis [61, 62]. However, only a limited amount of cfDNA, possibly less than 10 ng, can be obtained from standard blood test plasma samples, posing fundamental challenges for many methods to detect cfDNA methylation signatures accurately. Considering the clinical significance of cfDNA methylation as well as the scarcity of clinically feasible cfDNA, we assessed the performance of DMN– through benchmark experiments with input amounts of 1 ng, 0.5 ng, and 0.1 ng of plasma cfDNA from one CRC patient and one healthy control from the same cohort recruited by UCMC. By comparing normalized sequencing coverage as a hypomethylation enrichment score for each input, we found a high correlation across all comparisons and high reproducibility even at 0.1 ng, suggesting the robustness and reliability of DMN– in low-input cfDNA methylation profiling (Fig. 5a).

To further assess the performance of DMN– compared with existing methods known for their low-input application, we compared it with data generated from 1 ng and 0.1 ng of human cfDNA from the study of Cui et al. (2024) via three 5mC-seq methods: linear amplification-based bisulfite sequencing (LABS) [63], MethylC-seq [64] and EpiGnome (Illumina TruSeq DNA Methylation method). With an average of 17.3 million sequencing reads per sample, DMN– demonstrated a comparable mapping ratio across both input levels as did all three methods which have an average of 44.9 million reads per library (Fig. 5b) as well as genome-wide repeats and LINE-1 elements (Additional file 1: Fig. S8d-e). Comparing the normalized sequencing coverage between the two input levels, DMN– also exhibited a comparable correlation as all three methods did (Fig. 5c), verifying the detection sensitivity and potential clinical utility of DMN–. Additionally, by comparing CRC and healthy control samples, we observed more distinct separation based on disease status rather than input level, indicating that DMN– captures more significant biological variances than technical variances (Fig. 5d). Hence, these results validate the high sensitivity and robustness of DMN– in detecting true biological signals in input-limited clinical samples, reinforcing its potential as a novel approach for cancer detection and biomarker discovery.

## Discussion

Increasing interest in the biological and clinical significance of 5mC has driven rapid development of various 5mC-seq methods which, however, have various limitations such as high sequencing costs and limited single-base resolution, restricting clinical use. To address this, we developed DMN-seq utilizing the DNA glycosylase DME which specifically excises 5mC to produce single-strand breaks proportional to methylation density. Combined with optimized high-throughput sequencing, DMN+, the unique experimental design of DMN-seq, allows accurate, quantitative, and single-base 5mC detection while reducing sequencing costs through selective methylated DNA enrichment, highlighting its advantages as a novel 5mC sequencing method.

While DMN + provides a cost-effective strategy for quantitative 5mC mapping through methylation enrichment, DMN–, the alternative design of DMN-seq applied to profile the underexplored hypomethylated regions through depletion of hypermethylation regions presents a critical advancement. As global hypomethylation is typically reflected in regions with high sequence similarities, bisulfite-based methods which are typically used to characterize hypermethylated regions further reduce sequence diversity and cause mapping inaccuracies. DMN–, in contrast, addresses this by selectively enriching hypomethylated DNA. While technologies such as MRE-seq, mTAG-seq [65], and ACTIVE-seq [66], were also designed to enrich unmethylated CpG and, in principle, should be effective for detecting unmethylated sites especially in genomic repetitive regions, DMN– showed superior performance in higher mapping ratios across genome-wide repeats and LINE-1 and greater specificity for truly unmethylated DNA than MRE-seq. Additionaly, unlike mTAG-seq and ACTIVE-seq that rely on engineered methyltransferases to label unmethylated CpGs which may be influenced by sequence context and cause nonunifomred CpG enrichment, DMN– utilizes the glycosylase DME that has been validated to cleave 5mC sites in a motif-independent manner. Furthermore, mTAG-seq and ACTIVE-seq enrich for DNA fragments containing at least one unmethylated CpG but may retain internal 5mC sites that are indistinguishable from unmethylated C. In contrast, DMN– introduces cleavage at all 5mC sites, removing methylated fragments and enriching only for DNA truly depleted of 5mC. Therefore, DMN– enables more specific detection of unmethylated regions with minimum risk of misinterpreting methylated sites.

We thus successfully applied DMN– in CRC gDNA to investigate DNA global hypomethylation and enrichment of upregulated, CRC-associated pathways. Importantly, DMN– identified statistically significant hypomethylated loci as potential clinical biomarkers, highlighting its utility for cancer biomarker discovery. Additionally, we also identified regions that were significantly hypomethylated in normal colon tissue, suggesting that these regions are hypermethylated in CRC tumors. Further analysis of these regions validated known hypermethylated biomarkers for CRC, such as the FDA-approved *SEPT9*. These highlight the potential of DMN– to capture both hypo- and hypermethylated signatures.

Lastly, DMN– efficiently profiled hypomethylation signatures in cfDNA with inputs as low as 0.1 ng, further demonstrating its high sensitivity and reliability for large-scale comprehensive methylome analysis in clinical settings. The high mapping ratio and accuracy of DMN– also make it an ideal method to validate and quantify known biomarkers when coupled with targeted amplification methods [67]. This approach would further reduce the cost and barriers associated with the clinical applications.

## Conclusions

DMN-seq provides a novel and effective method to detect 5mC at single-base resolution with reduced sequencing cost. It also presents a highly sensitive and robust approach to differentiate hypomethylated regions in tumor gDNA and low-input cfDNA, creating new avenues to discover and investigate hypomethylated regions as biomarkers for cancer and other disease detection and management.

## Methods

### Protein expression and purification

PLM 302 DMEΔN677ΔIDRI::lnk was expressed and purified following the procedure described in Mok et al*.* (2010) [14]. In brief, the DME construct was transformed into the *E. coli* Rosetta2 (DE3) strain (Novagen). The expressed DME protein was first purified through a 5-mL HisTrap FF column (GE Healthcare), from which the collected fractions were digested with 15 U (7.5 µl) of PreScission Protease (GE Healthcare). The digested fractions were then purified through a 5-mL HiTrap Heparin HP column (GE Healthcare). The final gel filtration was performed on a HiLoad 16/600 Superdex 200-pg column (GE Healthcare).

### Protein reactivity test on 5mC and 5hmC

100 ng of 82 bp dsDNA 5mC oligo and 82 bp dsDNA 5hmC oligo (IDT, Additional file 1: Table S1), respectively, were treated in 1 × DME reaction buffer (80 mM Tris–HCl, pH 8, 200 mM NaCl, 200 mM EDTA, 1 mM dithiothreitol (DTT), 400 μg/mL BSA) and a reaction volume of 30 µl with 0, 125, 250, 500, 750 and 1000 ng of DME, and the treatment reaction was carried out at 37 °C for 2 h. After purification with a DNA Clean & Concentrate kit (Zymo Research), the oligos were eluted in 20 µl of water and mixed with 20 µl of Novex 2 × Tris–borate-EDTA (TBE)-Urea Sample Buffer (Invitrogen) to run on 10% Novex TBE-Urea Gel (Invitrogen) at constant 180 V for 80 min. The gel was stained with 2 µl of SYBR Gold Nucleic Acid Gel Stain (Invitrogen) in 25 ml of TBE buffer at room temperature for 15 min. The gel was then imaged using the ChemiDoc MP imaging system (Bio-Rad) at the University of Chicago BioPhysics Core Facility.

### mESC culture and gDNA extraction

The mESC cell line ES-E14TG2a was purchased from ATCC and grown on gelatin-coated plates in complete DMEM (Invitrogen Cat. No. 11995) supplemented with 15% vol/vol FBS (Gibco), 1% penicillin/streptomycin (Gibco), 1.25 × nucleoside (MilliporeSigma), 62.5 mM 2-mercaptoethanol (Thermo Fisher Scientific), 1.25 × nonessential amino acids (Gibco), 104 U/ml leukemia inhibitory factor (LIF) (MilliporeSigma), 0.289 mg per 500 ml of PD0325901 (STEMCELL Technologies), 0.83 mg per 500 ml of CHIR99021 (STEMCELL Technologies), and 5 mg ml − 1 Plasmocin Prophylactic (Invitrogen). The cells were cultured at 37 °C with 5.0% CO2 and passaged every 2 days. Authentication and mycoplasma contamination testing were performed within the lab to confirm cell identity and purity. After the cells were harvested via centrifugation for 3 min at 1000 × *g*, gDNA was extracted with a PureLink Genomic DNA Mini Kit (Invitrogen) following the manufacturer’s protocol.

### CRC patient recruitment and collection of tissue and plasma samples

Patient samples and unidentified data were supplied under The University of Chicago protocol (IRB#10–209-A). The protocol involves data and sample collection from newly diagnosed patients with nonmetastatic colorectal cancer and noncancer controls undergoing colonoscopies that are negative at the University of Chicago Medical Center (UCMC). Blood from patients with CRC was collected prior to surgical operation via EDTA-containing vials and processed within six hours for plasma. Biopsies of primary tumors and adjacent normal-appearing colonic mucosa were also collected and stored in RNA*later* solution (Thermo Fisher). Plasma and tissues in RNA*later* were stored at −80 °C before gDNA and cfDNA extraction. gDNA was extracted via an AllPrep DNA/RNA/miRNA Universal Kit (Cat. No. 80244) following the manufacturer’s protocol. In brief, the tissue was removed from the RNAlater solution for lysis and homogenization, and the DNA was collected by transferring the lysate through an AllPrep DNA Mini spin column (Qiagen). cfDNA was extracted from 1 mL of plasma via QIAamp Circulating Nucleic Acid Kit (Qiagen) following the instructions of the protocol.

### Library preparation

#### Library preparation starting with λ-DNA and mESC gDNA for methylated DNA enrichment in DMN+ 

200 ng of λ-DNA (NEB) or mESC gDNA supplemented with 0.1% (w:w) λ-DNA were fragmented with NEBNext dsDNA Fragmentase at 37 °C for 20 min and purified with a DNA Clean & Concentrate Kit (Zymo Research). Fragmented λ-DNA was added with a 1% (w:w) 200 bp dsDNA oligo (IDT, Additional file 1: Table S1) and a 1% (w:w) 164 bp dsDNA oligo as spike-ins (IDT, Additional file 1: Table S1). The fragmented mESC gDNA/λ-DNA mixture was supplemented with 0.01% (w:w) 200 bp dsDNA oligo and 0.01% (w:w) 164 bp dsDNA oligo as spike-ins. The DNA mixture or cfDNA was then added to reagents from the NEBNext Ultra II End Repair/dA-Tailing Module (NEB) and incubated sequentially at 20 °C for 30 min and 65 °C for 30 min. Using the NEBNext Ultra II Ligation Module (NEB), 1/4 of the DNA mixture or 5 ng of cfDNA, which serves as the UBS-seq control samples, was ligated with 15 µM methylated NEBNext adaptor (NEB), while the rest was ligated with 15 µM customized DME dsDNA adaptor with phosphorothioate modifications (IDT, Additional file 1: Table S1) at 20 °C for 15 min. The ligated DNA samples were purified with 1.0 × AMPure XP beads and eluted in H_2_O.

The UBS-seq sample was subjected to UBS-1 conditions and desulphonated via DNA Methylation-Gold Kit (Zymo Research) following the UBS-seq procedure [68]. The other DNA mixture sample was split into ¼ as an untreated input sample to proceed directly to PCR and ¾ as a DME treatment sample. All the DME-treated samples were subjected to a series of enzyme digestions with endonuclease IV (NEB), lambda exonuclease (NEB), exonuclease III (NEB), and quick CIP (NEB) following the manufacturer’s protocols to remove fragments that were not successfully ligated to the DME dsDNA ligator and/or contained 5’-P, resulting in false positives. After purification with 1.0 × AMPure XP beads, the DME treatment sample from λ-DNA or mESC gDNA was divided into 3 equal parts of 50 ng as 3 technical replicates. Subsequently, in 1 × DME reaction buffer (80 mM Tris–HCl, pH 8, 200 mM NaCl, 200 mM EDTA, 1 mM dithiothreitol (DTT), 400 μg/mL BSA) and a reaction volume of 30 µl, 50 ng samples were treated with 400 ng of DME, and the treatment reaction was carried out at 37 °C for 2 h. After purification with a DNA Clean & Concentrate kit (Zymo Research), each replicate was treated with endonuclease IV (NEB) and exonuclease III (NEB), sequentially, following the manufacturer’s protocols and heat denatured at 95 °C for 5 min to remove the fragments generated from DME excision that did not contain 5’-P.

5mC-containing fragments with 5’-P in each replicate were then ligated to a customized DME ssDNA adaptor (IDT, Additional file 1: Table S1) via the terminal deoxyribonucleotidyl transferase-assisted adenylate connector-mediated ssDNA (TACS) ligation method following the procedure described by Miura et al. [69]. Briefly, each DNA sample was heat denatured in a 23 μl reaction mixture containing 10 μl of 2.5 × TACS reaction buffer (125 mM HEPES–KOH, pH 7.5; 12.5 mM MgCl_2_; 1.25% (v/v) Triton-X100; 50% (w/v) polyethylene glycol (PEG) 8000); and 1 μl of 10 mM ATP and 5 μM DME ssDNA adaptor at 95 °C for 3 min. The mixture was then supplemented with 1 µl of 100 U/μl CircLigase II ssDNA ligase (Biosearch Technologies) and sequentially incubated at 37 °C for 30 min, 65 °C for 1 h and 95 °C for 5 min. After purification with 1.2 × Ampure Beads, DME-treated triplicates were amplified via customized DME P5 primer (IDT, Additional file 1: Table S1) and P7 primers from NEBNext Multiplex Oligos for Illumina (NEB) in NEBNext Ultra II Q5 Master Mix (NEB). The NEBNext Universal PCR Primer for Illumina and P7 primers NEBNext Multiplex Oligos for Illumina (NEB) were used to amplify the untreated input sample in NEBNext Ultra II Q5 Master Mix (NEB), and the UBS-seq sample was amplified in KAPA HiFi Uracil + DNA polymerase (Roche). Finally, all the libraries were cleaned with 2 rounds of 0.9X AMPure XP beads, and their quality was examined via a high-sensitivity fragment analyzer at the University of Chicago Genomics Core Facility. λ-DNA libraries were sequenced at PE150 on the Illumina NextSeq 1000/2000 platform at the University of Chicago Single-Cell Immunophenotyping Core, and mESC gDNA libraries were sequenced with the Illumina NovaSeqX platform.

#### Library preparation starting with mESC gDNA, CRC tissue gDNA, adjacent healthy tissue gDNA, and CRC patient and healthy control cfDNA for unmethylated DNA enrichment in DMN–

A total of 10 ng of gDNA from mESC and CRC patient cohort tumor tissue and adjacent healthy colon tissue was used as the untreated sample, whereas 40 ng was used as the DME-treated sample. Three technical replicates were used for mESCs, while 1 was used for CRC cohort gDNA. All gDNA samples were prepared, fragmented, and end-repaired in the same way as those used for methylated DNA enrichment. 1 ng, 0.5 ng., and 0.1 ng of cfDNA from CRC patients and healthy controls was used as the untreated sample, whereas 2 technical replicates of the same input level were used as DME-treated samples. The cfDNA samples were directly end-repaired without fragmentation. All DNA samples were then ligated to individual NEXTFLEX Unique Dual Index Barcodes (Revvity) following the recommended concentration on the basis of the input amount. After 0.8 × Ampure bead purification, all the DME-treated samples were pooled on the basis of DNA type and concentration, *i.e.,* 40 ng of mESC samples were pooled together, 40 ng of CRC and healthy gDNA samples were pooled together, and cfDNA duplicates were pooled at each input level. All pooled samples were primer extended using R2 primer (IDT, Additional file 1: Table S1) and Q5 Hot Start High-Fidelity 2X Master Mix (NEB) and cleaned with 1.2 × Ampure beads. DME treatment was conducted as described previously, followed by heat denaturation at 95 °C for 5 min and 0.8 × Ampure bead purification. All the DME-treated and untreated samples were then amplified in Q5 Hot Start High-Fidelity 2X Master Mix (NEB) and NEXTFLEX® Primer Mix 2.0 (Revvity). Finally, all the libraries were cleaned with 2 rounds of 0.8 × AMPure XP beads, and their quality was examined via a high-sensitivity fragment analyzer at the University of Chicago Genomics Core Facility. All libraries were then sequenced at PE150 with the Illumina NovaSeqX platform at the University of Chicago Genomics Core Facility.

#### Library preparation starting with CRC tissue gDNA and adjacent healthy tissue gDNA using RRBS method

RRBS libraries were processed using Zymo-seq RRBS Library Kit (Zymo Research) following the manufacturer’s protocol. Briefly, DNA was digested with MspI for 4 h, followed by adapter ligation, bisulfite conversion, and index‑primer amplification. All the libraries were cleaned with 2 rounds of 0.8 × AMPure XP beads, and their quality was examined via a high-sensitivity fragment analyzer at the University of Chicago Genomics Core Facility. All libraries were then sequenced at PE100 using NovaSeqX platform at the University of Chicago Genomics Core Facility.

## NGS data processing and analysis

### DMN-seq of methylated DNA enrichment in DMN + 

After trimming the TruSeq sequencing adapters from the 3′ ends of read 1 and read 2 via the Cutadapt (v4.9) tool, low-quality, short reads or reads without 3 consecutive A from the beginning were filtered out. The clean reads were then aligned to the spike-in sequences (164 bp and 200 bp synthetic dsDNA spike-ins in Additional file 1: Table S1 and λ-DNA in GenBank J02459.1) and reference genomes (mouse reference mm10 for mESC samples) via HISAT2 (v2.2.1). The sorted mapped reads in the mESC samples were then processed to remove PCR duplicates via the Picard tool (3.1.1). Finally, the number of signals was calculated by counting the number of starts of reads at each genomic site.

### DMN-seq of unmethylated DNA enrichment in DMN–

After trimming the TruSeq sequencing adapters from the 3′ ends of read 1 and read 2 via the Cutadapt (v4.9) tool, low-quality and short reads were filtered out. The clean reads were then aligned to the spike-in sequences (the 164 bp and 200 bp synthetic dsDNA spike-ins in Additional file 1: Table S1 and λ-DNA in GenBank J02459.1) and reference genomes (mouse reference mm10 for mESC samples; human hg38 for cfDNA samples and CRC samples) via HISAT2 (v2.2.1). The sorted mapped reads in the mESC, cfDNA and CRC samples were then processed to remove PCR duplicates via the Picard tool (3.1.1). Finally, the reads were summed via the featureCounts (v2.0.8) tool for each TSS upstream 2000 bp region downloaded from the UCSC genome browser.

### RRBS analysis of CRC tissue gDNA and cfDNA

After trimming the TruSeq sequencing adapters from the 3′ ends of read 1 and read 2 via the Cutadapt (v4.9) tool, low-quality and short reads were filtered out. The clean reads were then aligned to the human hg38 reference genome via HISAT3N (v2.2.1-3n-0.0.3) with the ‘–no-splice-alignment' argument. The sorted mapped reads in the cfDNA and CRC samples were then processed to remove PCR duplicates via the Picard tool. Finally, the number of converted and unconverted reads at all C sites was counted using the hisat-3n-table command, and the methylation ratio was calculated as the number of unconverted reads divided by total coverage.

## Supplementary Information

Additional file 1: DMN-seq Supplementary Figures. Fig. S1. Detailed schematics of DMN-seq for methylated and unmethylated DNA enrichment. Fig. S2. Validation of DMN-seq for unmethylated DNA enrichment in spike-ins. Fig. S3. Validation of DMN-seq for 5mC detection in mESC gDNA. Fig. S4. Validation of DMN-seq for hypomethylation profiling in spike-ins and genomic DNAs. Fig. S5. Comparison of DMN-seq and MRE-seq by mapped ratio in the genome-wide repetitive region and hypomethylation enrichment. Fig. S6. Application of DMN-seq for hypomethylation profiling in CRC tumor and healthy tissue. Fig. S7. Application of DMN-seq for LINE-1 region biomarker identification in CRC tumor and healthy tissue. Fig. S8. Application of DMN-seq in ultralow-input cfDNA. Table S1. Synthetic dsDNA oligo sequence information. Table S2. Demographic and clinical information of the recruited CRC patients.

## Data Availability

Sequencing data have been deposited in the NCBI Gene Expression Omnibus (GEO) under accession codes GSE309434 [70], GSE312185 [71], and GSE 312186 [72]. Previously published sequencing data that was reanalyzed here is available under accession code GSE186007 [73]. Mapping and analysis scripts are available at GitHub repository under MIT license (https://github.com/yangli04/DMN-seq) [74] and Zenodo (https://doi.org/10.5281/zenodo.17211282) [75].
