# Supplementary material for: DMN-seq enriches DNA hypomethylated regions for biomarker discovery using 5-methylcytosine glycosylase
Source: Genome Biol. 2026 Feb 11;27:139. doi: 10.1186/s13059-026-03991-6 (PMC13097799; doi:10.1186/s13059-026-03991-6)
Supplement: Supplementary file 1 — Additional file 1: DMN-seq Supplementary Figures. Fig. S1. Detailed schematics of DMN-seq for methylated and unmethylated DNA enrichment. Fig. S2. Validation of DMN-seq for unmethylated DNA enrichment in spike-ins. Fig. S3. Validation of DMN-seq for 5mC detection in mESC gDNA. Fig. S4. Validation of DMN-seq for hypomethylation profiling in spike-ins and genomic DNAs. Fig. S5. Comparison of DMN-seq and MRE-seq by mapped ratio in the genome-wide repetitive region and hypomethylation enrichment. Fig. S6. Application of DMN-seq for hypomethylation profiling in CRC tumor and healthy tissue. Fig. S7. Application of DMN-seq for LINE-1 region biomarker identification in CRC tumor and healthy tissue. Fig. S8. Application of DMN-seq in ultralow-input cfDNA. Table S1. Synthetic dsDNA oligo sequence information. Table S2. Demographic and clinical information of the recruited CRC patients. [file 13059_2026_3991_MOESM1_ESM.docx]

**Supplementary Figures**

**DMN-seq enriches DNA hypomethylated regions for biomarker discovery using 5-methylcytosine glycosylase**

Yiding Wang^1,2,#^, Yang Li^2,3,#^, Chang Ye^2,4,#^, Iryna Irkliyenko^5^, Lu Gao^4,6^, Marc Bissonnette^6^, Qing Dai^2,4^, Weixin Tang^4^, Chuan He^2,4,7,8,*^

^1^Committee on Genetics, Genomics & System Biology, The University of Chicago, Chicago, IL, USA.

^2^Howard Hughes Medical Institute, The University of Chicago, Chicago, IL, USA.

^3^Pritzker School of Molecular Engineering, The University of Chicago, Chicago, IL, USA.

^4^Department of Chemistry, The University of Chicago, Chicago, IL, USA.

^5^University of California, Berkeley–University of California, San Francisco Graduate Program in Bioengineering, Berkeley San Francisco, CA, USA

^6^Department of Medicine, The University of Chicago, Chicago, IL, USA.

^7^Department of Biochemistry and Molecular Biology, The University of Chicago, Chicago, IL, USA.

^8^Institute for Biophysical Dynamics, The University of Chicago, Chicago, IL, USA.

^#^These authors contributed equally.

*Correspondence: chuanhe@uchicago.edu


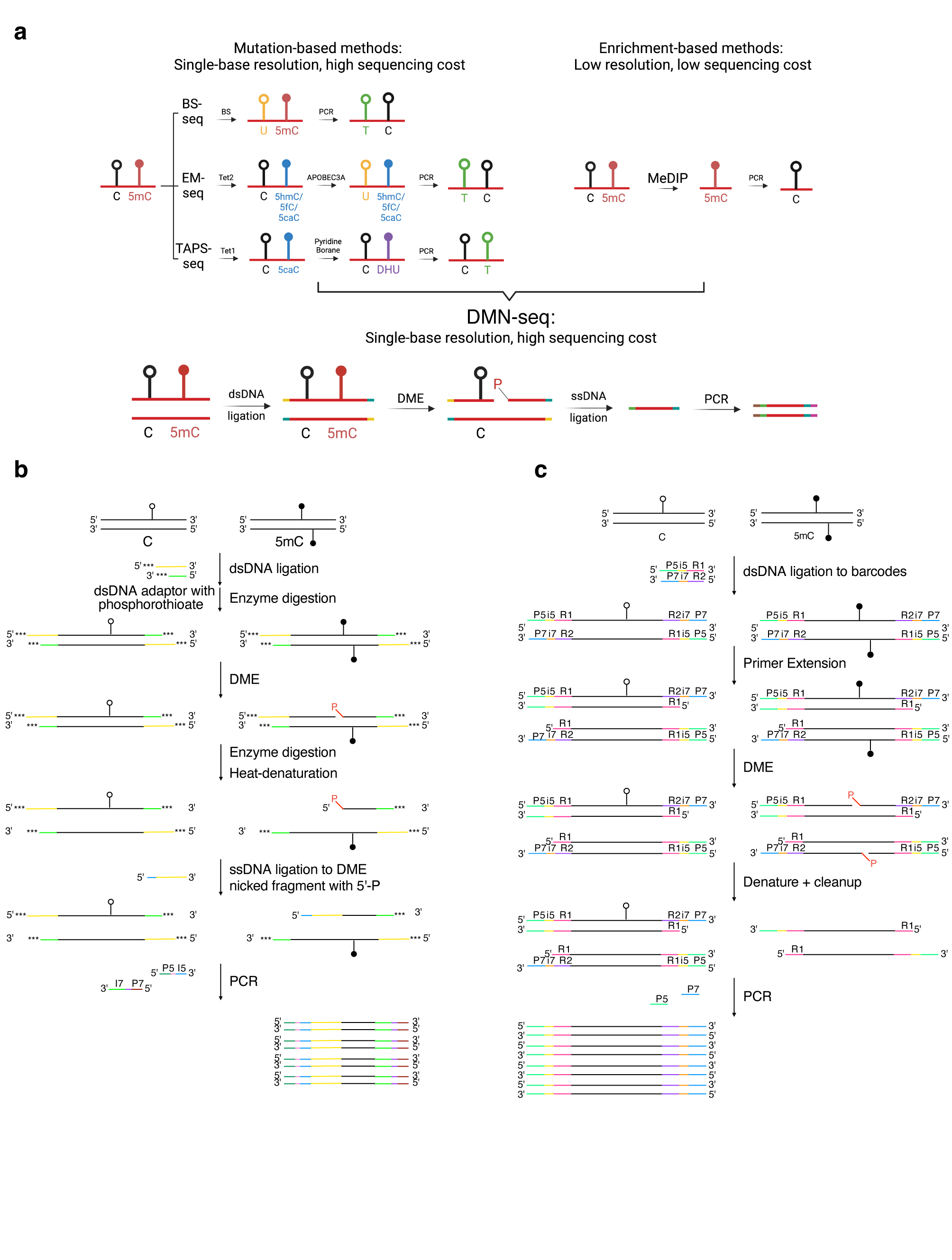


**Fig. S1. Detailed schematics of DMN-seq for methylated and unmethylated DNA enrichment.** **a**, Comparison of DMN-seq with established 5mC-seq methods including BS-seq, EM-seq, TAPS-seq, and MeDIP. **b**, Detailed schematic of DMN-seq for methylated DNA enrichment. **c**, Detailed schematic of DMN-seq for unmethylated DNA enrichment.


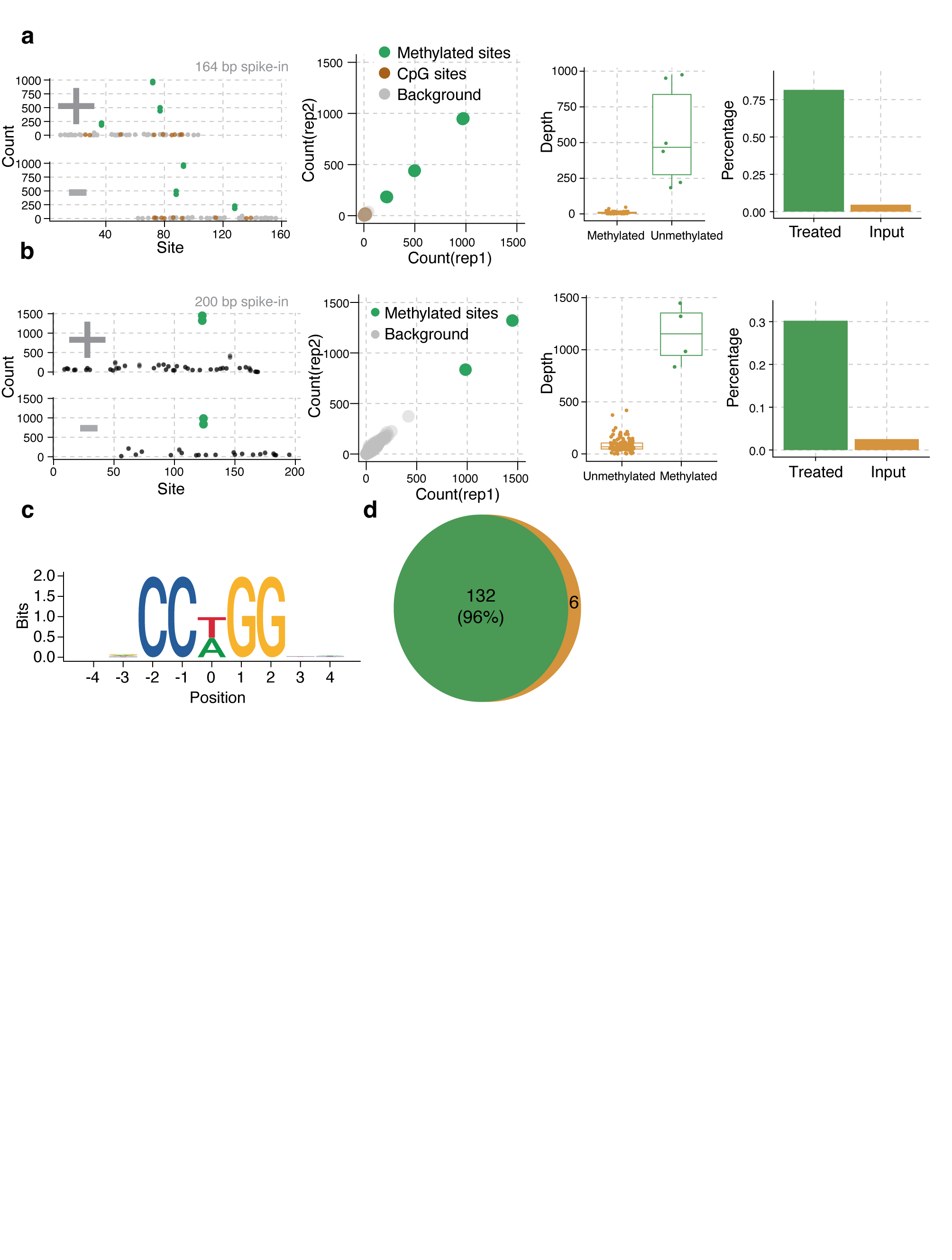


**Fig. S2. Validation of DMN-seq for unmethylated DNA enrichment in spike-ins.** **a**–**b**, Efficient enrichment and detection of known 5mC sites in synthetic 164 bp (**a**) and 200 bp (**b**) spike-ins. (Left) Read count distributions for 5mC sites detected by DMN-seq. (Middle) Bar plots showing the percentage of 5mC sites relative to all sites. (Right) Enrichment efficiency of 5mC in DME-treated samples versus untreated input. **c,** The consensus motif detected in λ-DNA aligns with the known DCM motif and displays minimal base bias in surrounding sequences. **d**, Comparison of DCM sites detected by DME (green) versus undetected sites (orange), potentially reflecting low methylation levels of some sites in partially methylated λ-DNA.


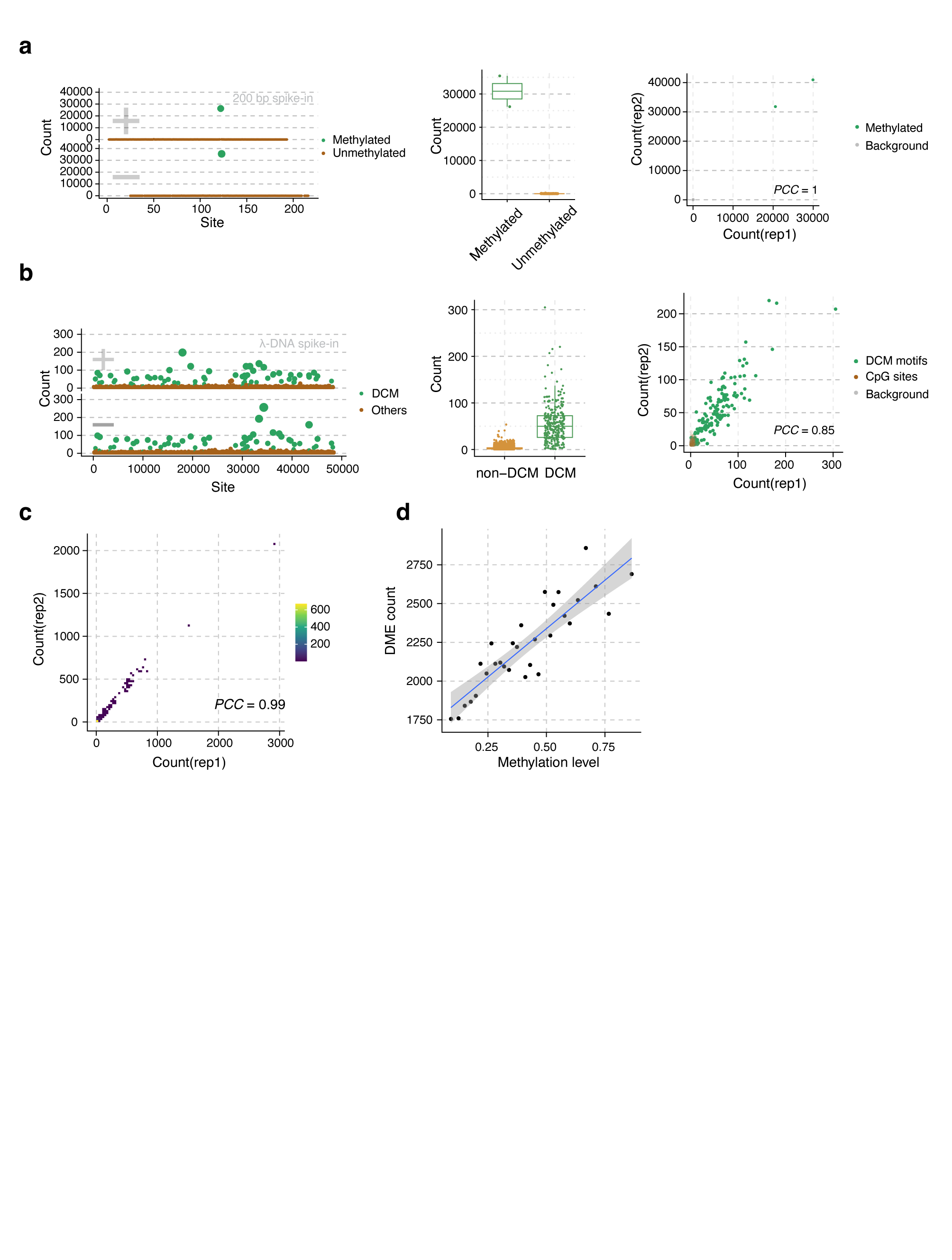


**Fig. S3. Validation of DMN-seq for 5mC detection in mESC gDNA.** **a**, Efficient enrichment and detection of known 5mC sites in synthetic 200 bp spike-ins. **b**, Detection of 5mC sites in λ-DNA spike-ins. (Left) Read count distributions of 5mC sites detected by DMN-seq. (Middle) Bar plots showing the read counts of 5mC sites compared to other sites. (Right) Scatter plot showing the correlation of read counts between two technical replicates. **c**, Correlation plot of DMN-seq read counts between two technical replicates in mESC gDNA. **d**, Significant correlation between methylation levels (detected by UBS-seq) and DMN-seq read counts (n = 30 bins).


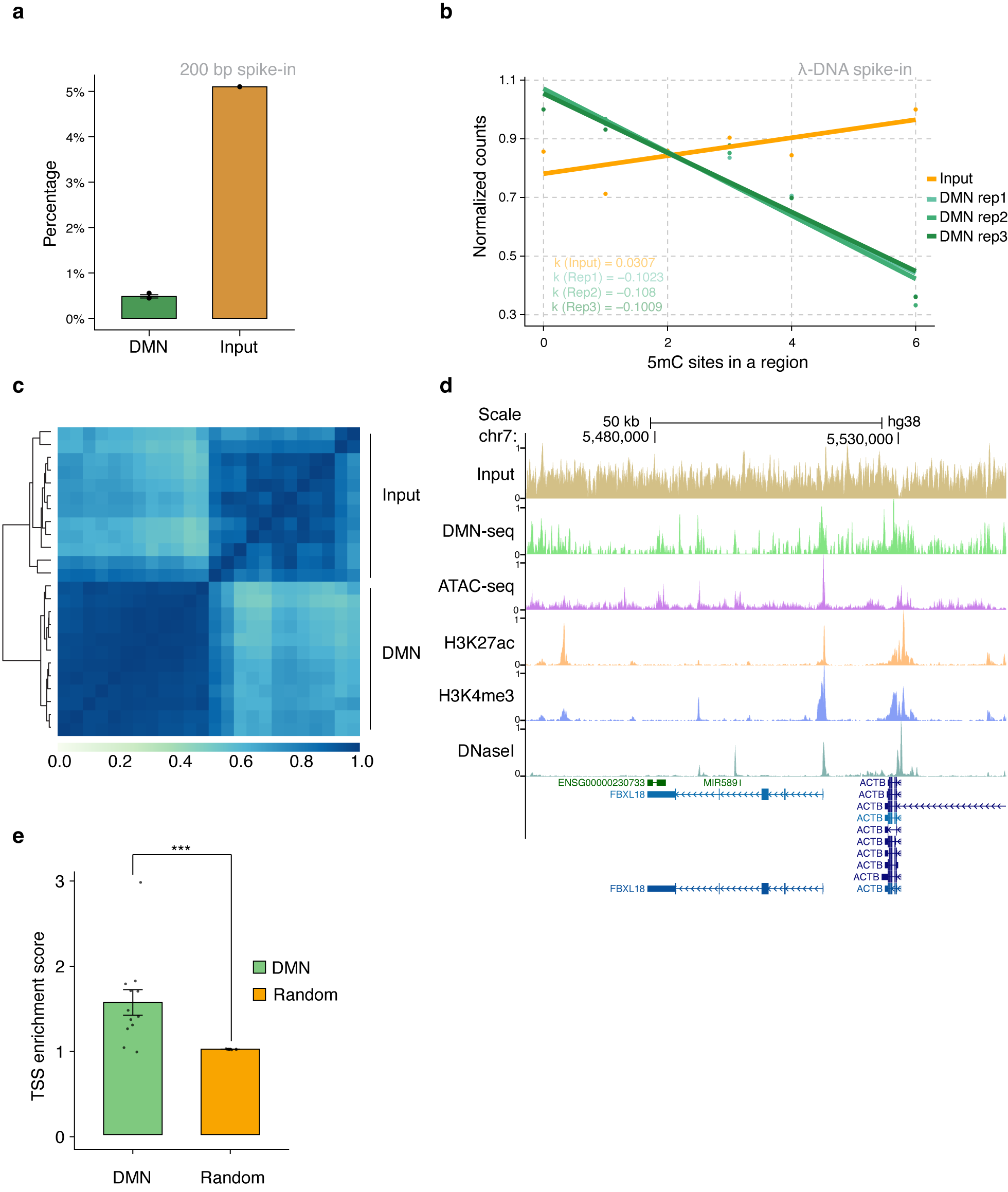


**Fig. S4. Validation of DMN-seq for hypomethylation profiling in spike-ins and genomic DNAs. a**, Averaged percentage of reads from 200 bp spike-in on DMN-seq and input samples. **b**, Plot of normalized read counts versus number of 5mC sites in 200 bp regions of λ-DNA spike-ins, showing that regions with more 5mC sites yield lower average read counts. **c**, Genome-wide Pearson correlation heatmap among DME-treated and input samples. Hierarchical clustering was performed using pairwise correlation coefficients calculated from 10-kb bins across the hg38 genome. DME and input samples form distinct clusters, reflecting the impact of DME treatment. **d**, UCSC genome browser snapshot illustrating DMN-seq signals in human gDNA alongside selected histone modifications, DNase I-seq, and ATAC-seq data at a representative locus. **e**, TSS enrichment scores calculated from DME peaks versus randomly shuffled peaks. A Wilcoxon test shows a significant difference (p = 0.0001006, n = 12 in each group), confirming TSS enrichment specificity.


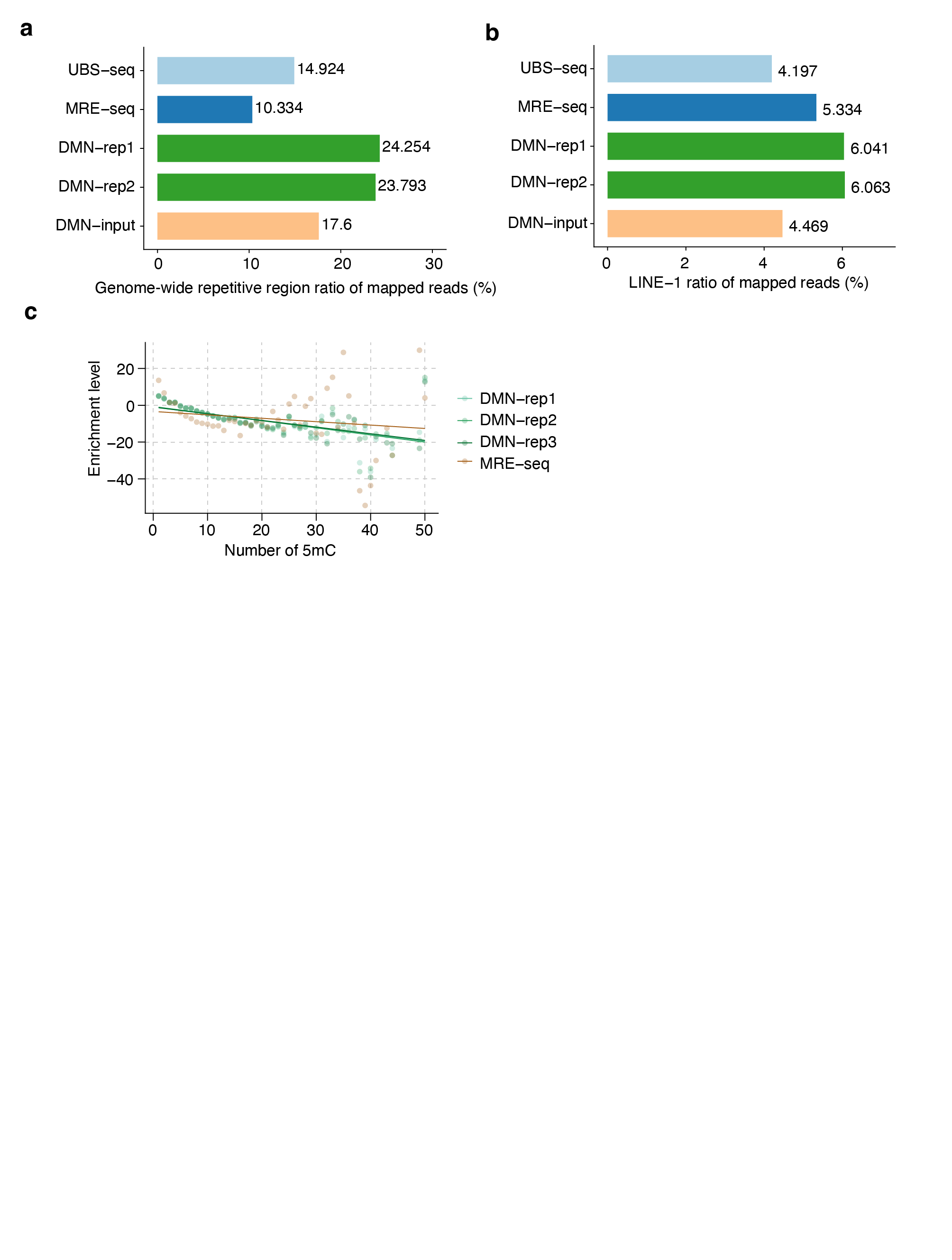


**Fig. S5. Comparison of DMN-seq and MRE-seq by mapped ratio in the genome-wide repetitive region and hypomethylation enrichment.**

**a**–**b,** Proportion of reads mapped to (**a**) genome-wide repetitive regions and (**b**) LINE-1 elements among all mapped reads in DMN-seq, UBS-seq, and MRE-seq from mESC gDNA (MRE-seq data: SRR414934, LINE-1 and repetitive region annotation were downloaded from UCSC genome browser). **c,** Enrichment of MRE-seq and DMN-seq in transcription start site (TSS) upstream 2,000 bp regions stratified by the number of 5mC sites. Scatter points represent the mean enrichment for regions with a given 5mC count, and lines indicate least-squares fits for each group.


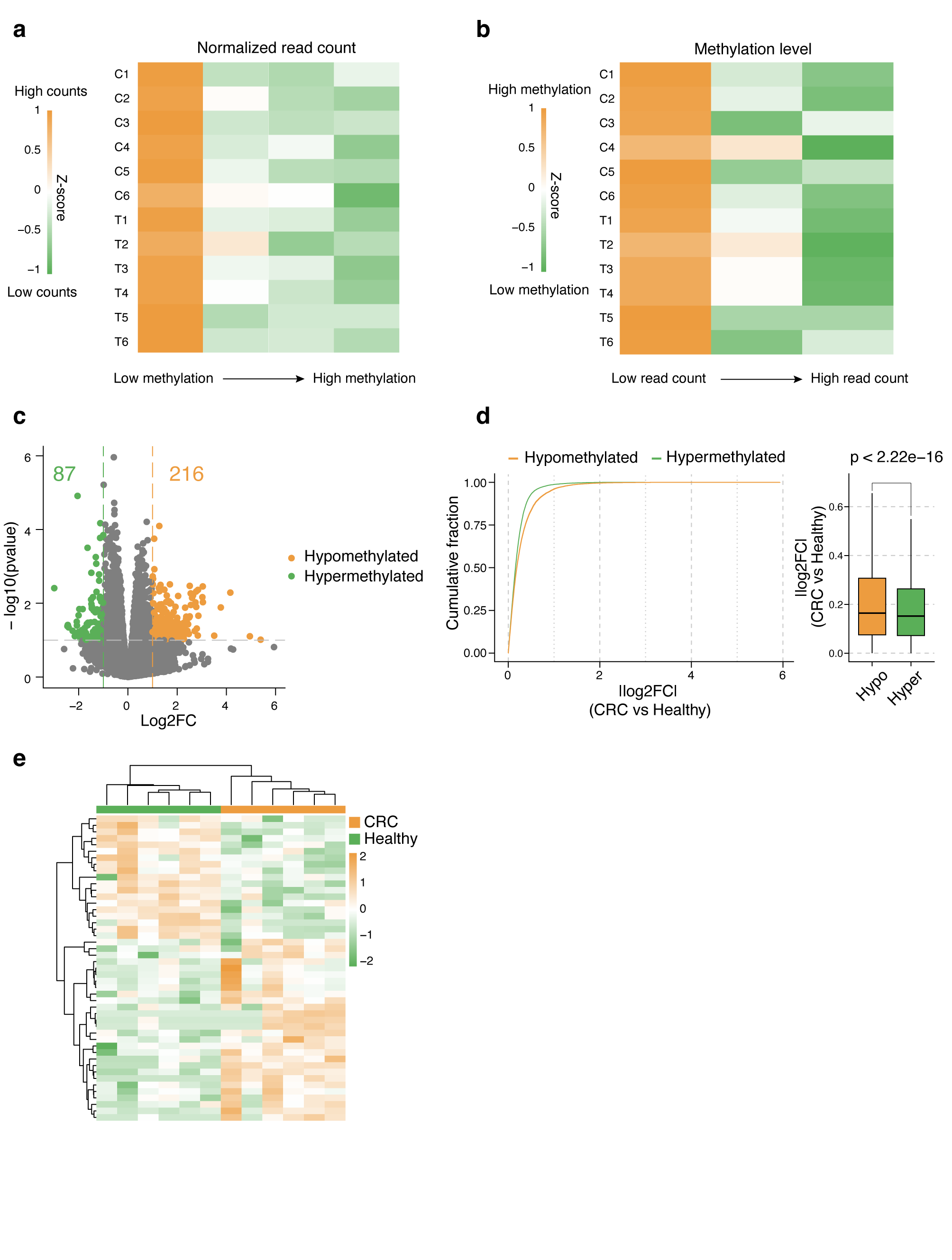


**Fig. S6. Application of DMN-seq for hypomethylation profiling in CRC tumor and healthy tissue. a**, Heatmap showing z-scores of normalized read counts across regions 2000 bp upstream of TSS, grouped by methylation level. Regions with higher methylation level exhibit lower read counts. Normalized counts were divided into equal-sized bins, ordered from highest to lowest. **b**, Heatmap showing z-scores of methylation levels across regions 2000 bp upstream of TSS, grouped by normalized read counts. Regions with higher read counts exhibit lower methylation levels. Normalized counts were divided into equal-sized bins, ordered from highest to lowest. **c**, Volcano plot displaying differentially expressed regions 2000 bp upstream of TSS across all samples. **d**, Distribution of absolute log2 fold changes (log2FC) for hypomethylated and hypermethylated regions comparing CRC and healthy tissues. e**)** Top differentially expressed regions identified by DMN-seq across all CRC and healthy samples.


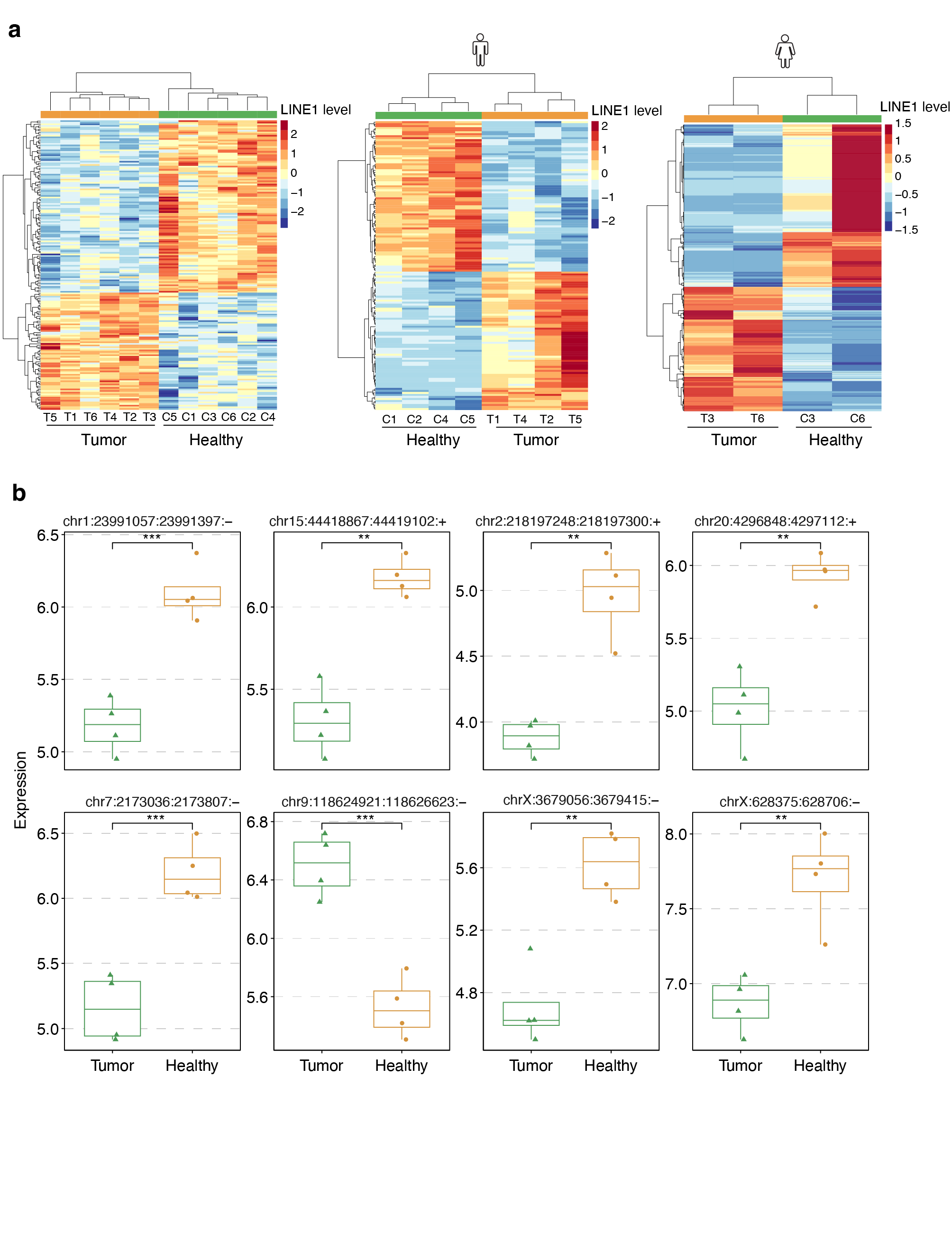


**Fig. S7. Application of DMN-seq for LINE-1 region biomarker identification in CRC tumor and healthy tissue. a**, Comparison of LINE-1 regions enrichment level between 6 CRC patients (T1-T6) and 6 control samples (C1-C6). Only regions with a significant differential enrichment with p-value less than 0.0001 were shown. The left, middle, and right shows the enriched LINE-1 in all samples, male group, and female group, respectively. The annotation of LINE-1 is downloaded from UCSC genome browser. **b**, Boxplot shows the identified top 8 differentially enriched LINE-1 elements in tumor and healthy control, which could serve as potential biomarkers for CRC diagnosis. The location of each region is labeled in the title of each panel. The p-values (t-test) shown on the figure indicate the significance of the differences between patients and healthy controls.


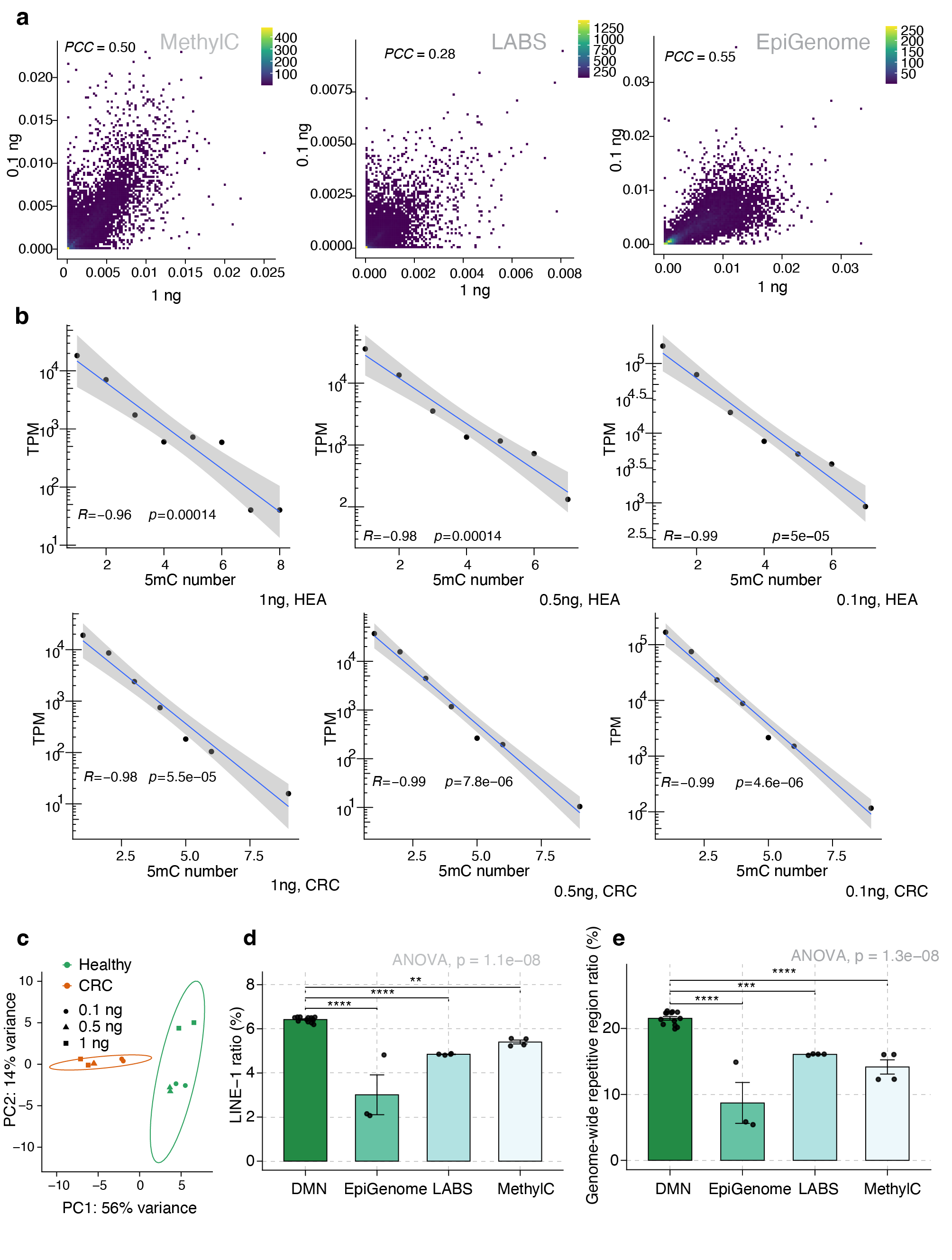


**Fig. S8.** **Application of DMN-seq in ultralow-input cfDNA**. **a**, Scatter plot showing average methylation levels across 2000 bp genomic regions, comparing MethylC-seq, LABS-seq, and EpiGenome methods using 1 ng and 0.1 ng cfDNA input. **b**, Scatter plots depict normalized read counts at TSS upstream 2000 bp regions versus the number of 5mC sites detected by LABS-seq in low-input cfDNA. **c**, PCA plot of cfDNA samples from CRC patients and healthy controls at input levels of 1 ng, 0.5 ng, and 0.1 ng, with 95% confidence ellipses highlighting group separation. **d**–**e** Comparison of the LINE-1 ratio (**d**) and genome-wide repetitive region ratio (**e**) in all mapped reads across different methods of cfDNA samples. Only samples with 1 ng cfDNA input are used. Bars show mean ± SEM of biological replicates. Individual points represent independent samples. Global differences were assessed by one-way ANOVA. Pairwise comparisons were performed with Dunnett’s post hoc test (two-sided), comparing each group against DMN, with multiplicity-adjusted p-values. *p < 0.05, **p < 0.01, ***p < 0.001, ****p < 0.0001; ns, not significant. The annotation for repetitive region and LINE-1 is downloaded from UCSC genome browser.

**Supplementary Tables**

**Table S1. Synthetic dsDNA oligo sequence information**

| **Name** | **Sequence** |
| --- | --- |
| 82bp dsDNA 5mC synthetic oligo (forward strand) | 5’- GTGACTGGAGTTCAGACGTGTGCTCTGCCTCCGATCTAGATGTGTAGTATCACGT5mCGCAGCTTGACCGCTCTAGTGACGGCT -3’ |
| 82bp dsDNA 5mC synthetic oligo (reverse strand) | 5’-AGCCGTCACTAGAGCGGTCAAGCTGCGACGTGATACTACACATCTAGATCGGAGGCAGAGCACACGTCTGAACTCCAGTCAC -3’ |
| 82bp dsDNA 5hmC synthetic oligo (forward strand) | 5’- GTGACTGGAGTTCAGACGTGTGCTCTGCCTCCGATCTAGATGTGTAGTATCACGT5hmCGCAGCTTGACCGCTCTAGTGACGGCT -3’ |
| 82bp dsDNA 5hmC synthetic oligo (reverse strand) | 5’-AGCCGTCACTAGAGCGGTCAAGCTGCGACGTGATACTACACATCTAGATCGGAGGCAGAGCACACGTCTGAACTCCAGTCAC -3’ |
| 200 bp dsDNA synthetic oligo (forward strand) | 5’- CCACACCACACCTATTCCAAAATTGACCACATAGTTGGAAGTAGAACTCTCCTCAGAACATGTAAAAGAACAGAAATTATAACAAACTATCTCTCAGACCACAGTGAACTCAAACTAGAGCT5mCGAGATTCAAAGCTTCTCAACTACATGGAAACTGAACAACCTGTCCCTGAATGACTACTGGGTACATAACAAGATCAACCCACAAATAAATCCCTTC -3’ |
| 200 bp dsDNA synthetic oligo (reverse strand) | 5’- GAAGGGATTTATTTGTGGGTTGATCTTGTTATGTACCCAGTAGTCATTCAGGGACAGGTTGTTCAGTTTCCATGTAGTTGAGAAGCTTTGAATCTCGAGCTCTAGTTTGAGTTCACTGTGGTCTGAGAGATAGTTTGTTATAATTTCTGTTCTTTTACATGTTCTGAGGAGAGTTCTACTTCCAACTATGTGGTCAATTTTGGAATAGGTGTGGTGTGG -3’ |
| 164 bp dsDNA synthetic oligo (forward strand) | 5’-  GTGAGTGGAGTTGAGAGGTGTGGTACGGTGACTCAGGTTTGTGCTCTTCCGATCTAGATGTGTAGTGCCATCCGAT5mCGCATATGCGAGTCACGTACATGCTACTGTCAGTACTGATGGACCTTTCT5mCGCAGTGGCGACTATGGTTGAGGGGTGTAGTGAGGGGT -3’ |
| 164 bp dsDNA synthetic oligo (reverse strand) | 5’- GTGAGTGGAGTTGAGAGGTGTGGTACGGTGACTCAGGTTTGTGCTCTTCCGATCTAGATGTGTAGTGCCATCCGAT5mCGCATATGCGAGTCACGTACATGCTACTGTCAGTACTGATGGACCTTTCT5mCGCAGTGGCGACTATGGTTGAGGGGTGTAGTGAGGGGT -3’ |
| dsDNA adaptor with phosphorothioate modifications | 5’-/5Phos/GAT CGG AAG AGC AC*A*C*G*T*C/3AmMO/-3’  5’-/5AmMC6/T*A*C* A*C*G AC GCT CTT CCG ATC T-3’ |
| 5’-ligation adaptor | 5’- biotin- CG ACC GAG ATC TAC ACT CTT TCC C TA CAC GAC GCT CTT CCG ATC TNN NNN rArArA -3’ |
| P5 primer | 5’- AAT GAT ACG GCG A CC GAG ATC TAC ACT CTT TCC C -3’ |
| R2 primer | 5’- TCTAGCCTTCTCGTGTGCAGACTTGAGGTCAGTG -3’ |

**Table S2. Demographic and clinical information of the recruited CRC patients.**

| Tumor ID | Accession # | STAGE | Lymph-Vascular Invasion | MSI/MSS | age | gender | race |
| --- | --- | --- | --- | --- | --- | --- | --- |
| 1 | S16-18826 | II | no | MSS | 55.8 | male | white |
| 2 | S18-8231 | II | no | MSS | 78.5 | male | white |
| 3 | S18-22006 | II | no | MSS | 60.1 | female | white |
| 4 | S16-14993 | II | no | MSS | 77.3 | male | black |
| 5 | S17-25897 | II | no | MSS | 63 | male | white |
| 6 | S18-10058 | II | no | MSI | 66.6 | female | white |
